# Genetic Modifiers of ALS: The Impact of Chromogranin B P413L in a Bulgarian ALS Cohort

**DOI:** 10.3390/genes15091197

**Published:** 2024-09-12

**Authors:** Ivan Tourtourikov, Tihomir Todorov, Teodor Angelov, Teodora Chamova, Ivailo Tournev, Vanyo Mitev, Albena Todorova

**Affiliations:** 1 Department of Medical Chemistry and Biochemistry, Medical University of Sofia, 1431 Sofia, Bulgaria; 2 Genetic Medico Diagnostic Laboratory Genica, 1612 Sofia, Bulgaria; 3 Department of Neurology, Faculty of Medicine, Medical University of Sofia, 1431 Sofia, Bulgaria; 4 Department of Neurology, Clinic of Nervous Diseases, Medical University of Sofia, UMBAL Aleksandrovska, 1431 Sofia, Bulgaria; 5 Department of Cognitive Science and Psychology, New Bulgarian University, 1618 Sofia, Bulgaria

**Keywords:** ALS, CHGB, P413L

## Abstract

This study investigated the role of the CHGB P413L variant (rs742710) in sporadic amyotrophic lateral sclerosis (sALS) within the Bulgarian population. We analyzed 150 patients with sALS (85 male and 65 female) for the presence of this variant, its potential impact on disease susceptibility, and age of onset. Genotyping was performed using PCR amplification and direct Sanger sequencing. Statistical analyses included comparisons with control data from GnomAD v2.1.1, one-way ANOVA, and Kaplan–Meier survival analysis. Results revealed a higher frequency of the minor T allele in patients with sALS compared to all control groups and a statistically significant increase in carrier genotypes compared to non-Finnish Europeans (χ^2^ = 15.4572, *p* = 0.000440). However, the impact on age of onset was less clear, with no statistically significant differences observed across genotypes or between carriers and non-carriers of the T allele. Kaplan–Meier analysis suggested a potential 2.5-year-earlier onset in T allele carriers, but the small sample size of carriers limits the reliability of this finding. Our study provides evidence for an association between the CHGB P413L variant and sALS susceptibility in the Bulgarian population, while its effect on age of onset remains uncertain, highlighting the need for further research in larger, diverse cohorts.

## 1. Introduction

Amyotrophic lateral sclerosis (ALS) is a progressive neurodegenerative disorder characterized by the degeneration of motor neurons in the brain and spinal cord, leading to muscle weakness, atrophy, and ultimately respiratory failure [1]. The pathogenesis of ALS is multifaceted, involving genetic, environmental, and lifestyle factors that contribute to its onset and progression [2]. The pathogenesis of ALS involves multiple genetic and molecular pathways. Key pathways implicated include protein homeostasis imbalance, RNA metabolism disorders, mitochondrial dysfunction, excitotoxicity, and disrupted intra-neuronal transport [3]. Recent advances in genomics have uncovered numerous genetic variants associated with ALS, offering new insights into the molecular mechanisms underlying this complex disease [4]. Mutations in genes such as C9orf72, SOD1, TDP-43, and FUS are central to ALS pathology, often leading to the accumulation of toxic proteins, RNA processing defects, and mitochondrial damage [5,6]. Specifically, TDP-43, a protein involved in RNA processing, shows nuclear depletion and cytoplasmic aggregation in ALS, disrupting neuronal function and contributing to neuromuscular junction (NMJ) dismantling and motor neuron loss [7]. Motor neuron hyperexcitability is another hallmark of ALS, characterized by increased neuronal activity even before clinical symptoms manifest. This hyperexcitability leads to mitochondrial dysfunction, energy metabolism disruption, and increased oxidative stress, which differentially affect motor neuron subtypes based on their metabolic needs and size [8]. Glutamate-mediated excitotoxicity, due to dysfunctional glutamate transporters, further exacerbates neuronal damage [9].

The typical age of onset for ALS varies between familial and sporadic cases, reflecting distinct genetic and environmental influences. Sporadic ALS, which constitutes approximately 90–95% of all ALS cases, typically presents around the age of 58–63 years. In contrast, familial ALS, accounting for the remaining 5–10% of cases, often has an earlier onset, usually between the ages of 47 and 52 years [10]. This earlier onset is primarily due to the presence of highly penetrant genetic mutations, such as those in the SOD1, TARDBP, and C9orf72 genes, which predispose individuals to the disease at a younger age. Understanding the causes of an earlier onset of symptoms is crucial for several reasons. Primarily, it aids in identifying the heterogeneity of the disease, which can influence prognosis and treatment strategies. Age of onset is often correlated with the genetic and environmental factors contributing to ALS, with younger onset cases frequently linked to genetic mutations, such as those in the SOD1 or C9orf72 genes, whereas older onset might indicate a stronger role for environmental factors [11]. Additionally, age of onset impacts survival rates; patients with younger onset ALS typically have a longer disease duration compared to those with later onset [12]. This knowledge is vital for designing targeted therapeutic interventions and for counseling patients regarding their prognosis. Moreover, understanding age-specific disease mechanisms can drive the development of age-tailored personalized therapies, potentially improving patient outcomes [13].

Single nucleotide polymorphisms (SNPs) can act as modifiers of age of onset in ALS, adding to the complexity of the genetic landscape of the disease. For example, the SNP rs12608932 in the KIFAP3 gene is associated with a slower disease progression and extended survival in sporadic ALS; carriers of the protective allele develop symptoms approximately 1.5 years later than non-carriers [14]. A genome-wide association study identified the rs10128627 variant as significantly associated with an earlier onset of ALS. This variant influences the expression of the NEAT1 gene, which regulates several ALS-associated proteins, including TDP-43. Patients with the rs10128627 variant develop ALS approximately 3.15 years earlier than those without this variant [15]. Another variant, rs4970944, located near the CTSS gene, was associated with a later onset based on the dosage of the A-allele, affecting the expression of CTSS in the brain [16]. 

Among the genes studied as modifiers of ALS, chromogranin B (CHGB) has emerged as a candidate of interest. CHGB is a neuroendocrine secretory protein involved in the regulation of neuropeptide production and neurotransmitter release [17]. Variants in the CHGB gene have been linked to various neurological disorders, suggesting a potential role in modulating neurodegenerative processes [18]. Notably, the P413L variant in CHGB has been proposed as a risk factor for ALS, although its impact on disease susceptibility and progression requires further investigation [19].

CHGB is a vital neuroendocrine secretory protein encoded by the CHGB gene, which is located on chromosome 20pter-p12. It belongs to the granin family, which includes other key proteins such as chromogranin A and secretogranin II. These proteins are predominantly found in secretory vesicles of neuroendocrine and endocrine cells, playing critical roles in the storage and release of hormones and neurotransmitters [20,21]. CHGB is primarily localized in the secretory granules of neuroendocrine and endocrine cells, including pancreatic β-cells and neurons. It is involved in the formation and trafficking of these granules, which are crucial for the storage and secretion of neurotransmitters and hormones [22]. CHGB is characterized by its acidic nature and extensive post-translational modifications, which include phosphorylation and proteolytic cleavage during neural differentiation [23]. The region where the P413L variant is located also constitutes a phosphorylated peptide, formed from the 368–417 residues of the protein [24]. The primary structure of chromogranin B allows it to aggregate and form secretory granules, which are essential for its storage function. Within these granules, chromogranin B undergoes proteolysis to produce several bioactive peptides, such as CCB [25], GAWK [26], PE-11 [27], chrombacin [28], and secretolytin [29]. Furthermore, the N- and C-terminal domains of the CHGB protein are conserved and have important regulatory activities, with the N-terminal domain being critical for binding to the inositol 1,4,5-trisphosphate receptor and the C-terminal domain acting as a calcium signaling amplifier [30,31].

A specific genomic variant c.1238C>T, resulting in the P413L substitution (rs742710), has been proposed as a risk factor and modifier of ALS onset, with initial studies suggesting an association with a 7-year earlier disease onset, particularly in French populations. It was hypothesized that this variant might influence the sorting of chromogranin B into secretory granules, potentially contributing to ALS pathogenesis [19]. However, subsequent investigations have yielded conflicting results. Studies in Italian and French cohorts failed to confirm an increased frequency of the P413L variant in patients with sporadic ALS compared to controls, nor did they find significant differences in age of onset between carriers and non-carriers [32,33]. The CHGB P413L variant’s role in ALS may also be influenced by sex-dependent effects, with studies indicating an earlier onset in female carriers potentially due to differences in chromogranin B expression regulated by sex-specific genetic elements [34]. A meta-analysis of several case-control studies concluded that there is no significant association between the P413L variant and ALS risk or age of onset [35]. The Bulgarian population, characterized by a unique genetic makeup and relatively stable demographic structure, presents an ideal cohort for studying the genetic factors associated with ALS [36]. 

The first study discussing the effect of the P413L variant showed that it caused abnormal sequestration of the CHGB protein within the ER–Golgi network, possibly leading to impaired sorting and maturation of CHGB into secretory granules [19]. Further characterizing the role of chromogranins in neurodegenerative disease, a study investigating their role in Parkinson’s disease (PD) discovered that peptide processing, mediated by CHGB, was compromised, leading to dysregulation of several related proteins and peptides [23]. CHGB has also been hypothesized to be a target of neoechinulin A, which modulates CHGB function to exert a protective effect against cytotoxic reactive nitrogen species [37].

This dimorphic protein exists in soluble and membrane-bound forms, enabling it to function across different cellular contexts. In the formation of dense-core secretory granules (DCSGs), CHGB facilitates the storage and regulated release of hormones and neurotransmitters, a process critical for cellular communication and homeostasis. Moreover, CHGB forms tetramer anion channels that are delivered to the cell surface upon granule exocytosis, maintaining ion balance during secretion [38]. CHGB channels are distributed among various intracellular membranes, including the endoplasmic reticulum (ER) and Golgi apparatus. They help in maintaining ion balance and the proper functioning of these organelles. Disruptions in these pathways are known to contribute to ALS pathology by causing ER stress and impairing protein folding [39]. Furthermore, TDP-43 mislocalization and aggregation in the cytoplasm of neurons lead to cellular dysfunction. Proper ion balance maintained by CHGB channels can influence these processes, as disruptions in ion homeostasis can exacerbate TDP-43 aggregation [40]. 

This study aims to explore the role of the CHGB P413L variant in ALS within the Bulgarian population, characterized by its unique genetic background. By analyzing the frequency of this variant in Bulgarian patients with ALS and its impact on disease onset and progression, we hope to gain deeper insights into the genetic factors influencing ALS, contributing to the development of personalized therapeutic strategies, and improving prognostic predictions.

## 2. Materials and Methods

### 2.1. Patients

The study group consisted of a total of 150 patients (Table 1) diagnosed with sporadic ALS, an expanded sALS cohort from our previous study [41]. The patients were diagnosed, referred for genetic testing, and received medical counseling and treatment from the team at the Clinic of Neurology, UMBAL “Alexandrovska”, Sofia, Bulgaria. Patients included in the study were above legal age (18 years) without pathogenic variants in ALS-related genes. No patients below the age minimum, patients without a clear diagnosis of ALS and patients harboring pathogenic variants in genes, known to cause ALS were included in this study. Data on the age of onset of the disease and the initial systemic involvement were collected from the Clinic of Neurology, UMBAL “Alexandrovska”, Sofia, Bulgaria. Blood draw was performed by certified phlebotomists at the Clinic of Neurology, UMBAL “Alexandrovska”, Sofia, Bulgaria. DNA extraction via desalting methods was performed using 6 ml venous blood samples at Genetic Medico Diagnostic Laboratory Genica, Sofia, Bulgaria. All patients provided signed informed consent before being enrolled in the study.

### 2.2. SNP Selection and Genotyping

Genotyping was performed at Genetic Medico Diagnostic Laboratory Genica, Sofia, Bulgaria. PCR amplification reactions were carried out in a 25 µL volume containing 50–100 ng of DNA, 0.2 µM of each dNTP, 0.2 µM of each primer, 0.1 U Taq polymerase, and 1x Pol buffer B with 2.5 mM MgCl2. The following primers were used: F: 5′-CCAGGAGGAATCTGAGGAGTC-3′ and R: 5′GTCCAGCTCTTTCCACGC-3′. Conditions used for the PCR reaction were as follows: 5 min initial denaturation at 95 °C, followed by 35 cycles at 95 °C for 30 s, 60 °C for 30 s, and 72 °C for 40 s; final extension was conducted at 72 °C for 5 min. Evaluation of the quantity and quality of the obtained amplification products was performed by visualization on agarose gel electrophoresis using a 3% agarose gel. Samples were analyzed in the presence of a molecular marker against which the length of the amplified fragment was read. The obtained product was sequenced by the direct Sanger sequencing method using the BigDye Terminator v.3.1 sequencing kit (Thermo Fisher Scientific, Waltham, MA, USA) and electrophoretic separation on a capillary sequencer (ABI Prism 3130 Sequence Genetic Analyzer). The obtained data were automatically processed by the ABI3130 Data Collection Software program and obtained in a ready form in the form of an electropherogram.

### 2.3. Statistical Analysis

Genotype and allele frequencies for rs742710 were compared between the patient cohort and data available from the Genome Aggregation Database (GnomAD, available at https://gnomad.broadinstitute.org/, accessed on 15 June 2024) v2.1.1 for 339 Bulgarian controls, 226 non-neuro controls, 24,133 non-Finnish European (NFE) controls, and 51,547 NFE non-neuro individuals [42]. The ALS patient group was compared against these control groups to identify any significant differences in genotype and allele distributions. The age of onset followed a normal distribution with a right skew, with 54% of the patients falling into the typical 55- to 70-year onset and the remaining 46% outside of this age range. One-way ANOVA was performed using genotype status or minor allele carrier status and age of onset using IBM SPSS Statistics for Windows version 25.0 (released 2017; IBM Corp., Armonk, New York, NY, USA) analysis software. Kaplan–Meier plots were generated using the lifelines library for the Python programming language by grouping the patients based on their genotype or allele carrier status for the whole cohort as well as split by sex and plotting the age of onset across the groups. 

## 3. Results

Both allele and genotype frequencies show a higher representation of the minor T allele in the patient cohort (Table 2).

**Figure 1:**
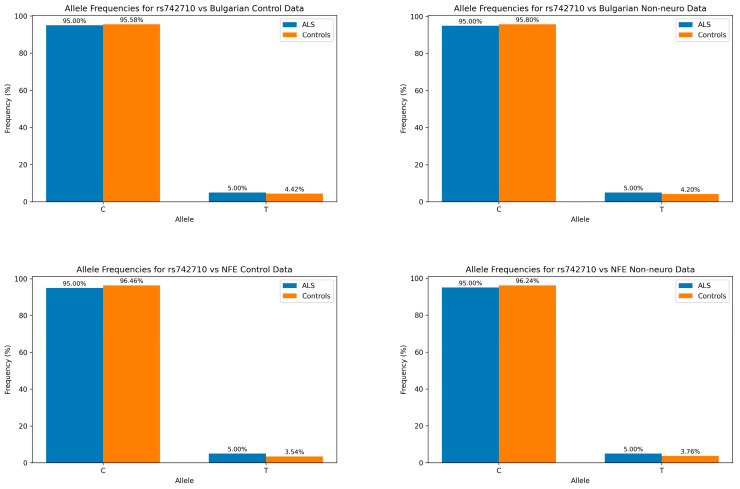
Bar charts depicting the allele frequencies between patients with ALS (blue) and control groups (orange). The minor allele of the rs742710 variant is present with a slightly elevated frequency in the patient group against all tested control populations.

The T allele exhibits a higher frequency in patients with ALS compared to all tested control groups without reaching statistical significance (*p*-values ranging from 0.328 to 0.818). The increase in T allele frequency in patients with ALS ranges from 0.58% to 1.24%. While this increase is marginal, it suggests a potential association between this allele and ALS susceptibility. A graphical representation of the allele frequencies is shown in Figure 1.

**Figure 2:**
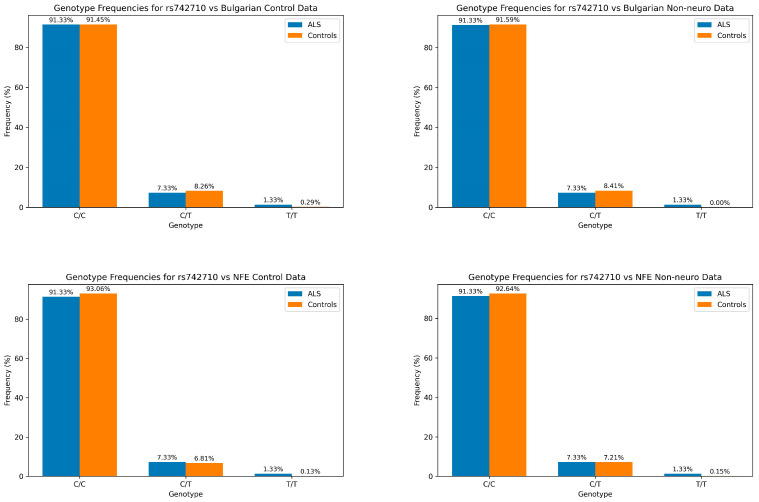
Bar charts depicting the allele frequencies between patients with ALS (blue) and control groups (orange). The carrier genotypes C/T and T/T for the rs742710 variant are slightly elevated compared to NFE controls and non-neuro individuals, while only the homozygous T/T genotype has a higher frequency compared to Bulgarian controls and non-neuro individuals.

Genotype distributions (Figure 2) showed a statistically significant difference from controls. Both C/T and T/T genotypes demonstrate significantly increased frequencies in patients with ALS relative to NFE control groups, with overall χ^2^ values ranging from 13.7498 to 15.4572 and *p*-values from 0.000440 to 0.00103. When comparing to Bulgarian control data, the genotype distributions did not reach a statistically significant level; however, the homozygous T/T genotype frequency is approximately ten times more frequent. The most statistically significant difference is observed in the comparison with NFE controls (χ^2^ = 15.4572, *p* = 0.000440). 

A one-way ANOVA analysis was performed to determine an effect on the age of onset. The analysis across the three genotypes revealed no statistically significant differences. The C/C group (*n* = 137) exhibited a mean age of onset of 58.35 years (SD = 12.44), while the C/T group (*n* = 11) and T/T group (*n* = 2) showed mean ages of onset of 55.27 years (SD = 12.11) and 59.50 years (SD = 14.85), respectively. The overall mean age of onset across all groups (*n* = 150) was 58.14 years (SD = 12.38) with no significant variation between groups (F(2, 147) = 0.324, *p* = 0.724). These findings suggest that the variant does not exhibit a significant influence on the age of onset when using an additive model in the studied population. The same analysis was performed for carriers of the T allele vs. non-carriers, as the two homozygous carriers were outliers (with age of onset 49 and 70). The wild-type group (*n* = 137) demonstrated a mean age of onset of 58.35 years (SD = 12.44), while the carrier group (*n* = 13) showed a mean age of onset of 55.92 years (SD = 11.96). The 95% confidence intervals for the mean age of onset were 56.25 to 60.45 years for the wild-type group and 48.69 to 63.15 years for the carrier group. The one-way ANOVA revealed no significant difference between groups (F(1, 148) = 0.455, *p* = 0.501). The overall results suggest that the P413L variant does not significantly impact the age of onset in the studied population.

To further investigate if the variant impacted the age of onset, we constructed Kaplan–Meier survival curves for both the genotypes and the alleles.

**Figure 3:**
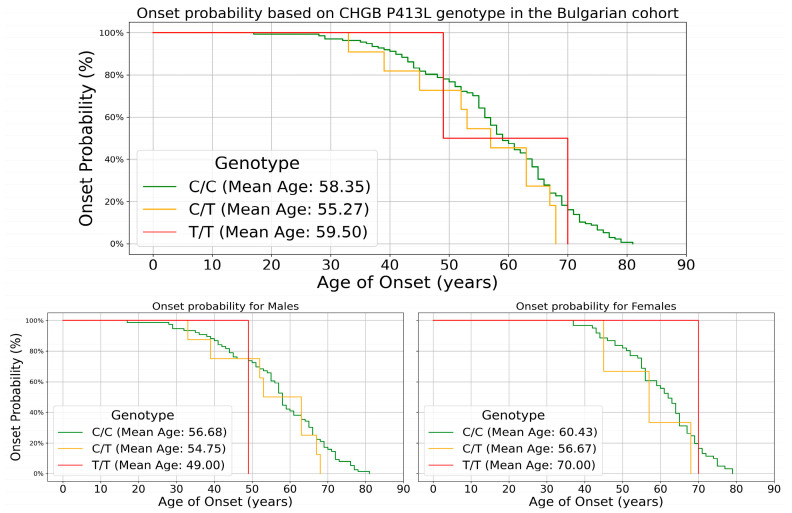
Kaplan–Meier survival curves for onset based on genotype for the entire patient cohort (**top**) and split by sex (**bottom**). Heterozygous carriers (orange) show an earlier onset regardless of sex, while homozygous T/T carriers (red) display an earlier onset only in males compared to controls (green).

At the genotype level, C/C homozygotes (*n* = 137) had a mean onset age of 58.4 years, C/T heterozygotes (*n* = 11) showed an earlier onset at 55.3 years, and T/T homozygotes (*n* = 2) had a mean onset of 59.5 years (Figure 3). Male patients showed an earlier onset compared to females across all genotypes.

**Figure 4:**
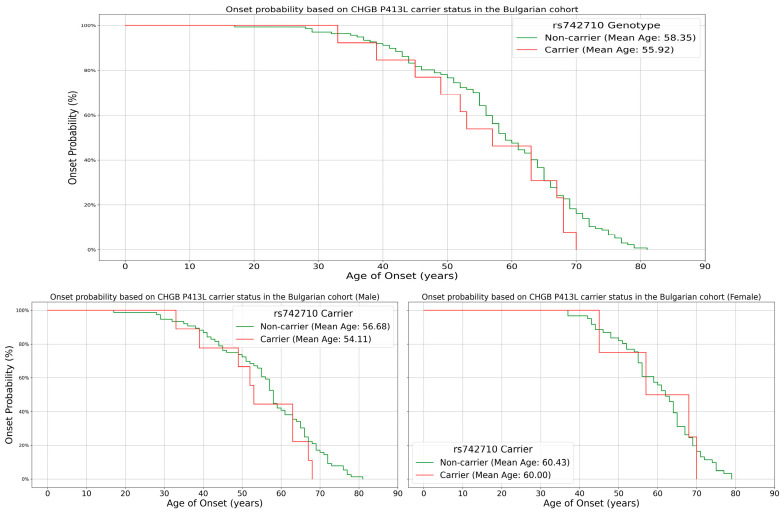
Kaplan–Meier survival curves for onset based on carrier status for the minor allele for the entire patient cohort (**top**) and split by sex (**bottom**). Carriers of the T allele (red) present with an earlier onset of the disease, with 2.43 years for the entire cohort, 2.57 years for males and 0.43 years for females compared to controls (green).

At the allele level, carriers of the T allele (*n* = 13, combining C/T and T/T) displayed a mean onset age of 55.9 years, suggesting a 2.5-year-earlier onset compared to non-carriers (C/C). This indicates a potential dosage effect, where a single T allele may be associated with earlier onset, but two T alleles might not further advance onset. The age range for T allele carriers (33–70 years) was narrower than for non-carriers (28–81 years), possibly suggesting a more constrained onset window (Figure 4). Interestingly, the effect of T allele carriage was more profound in males, with a 2.5-year-earlier onset compared to females (0.43 years). However, the limited number of T allele carriers necessitates cautious interpretation and further research with larger populations to confirm these trends.

## 4. Discussion

Understanding the role of the CHGB P413L variant in ALS necessitates a population-specific approach, as genetic diversity can influence disease manifestation and progression. Previous studies have highlighted the significance of population-specific genetic variants in elucidating the heterogeneity of ALS [43]. Our study investigated the role of the CHGB P413L variant (rs742710) in sporadic ALS within the Bulgarian population. The findings reveal several important insights into the potential impact of this variant on ALS susceptibility and disease onset. We observed a higher frequency of the genotypes harboring the risk T allele in our patients with ALS cohort compared to control groups. This difference was most pronounced when comparing our patients to non-Finnish European (NFE) controls, with a statistically significant increase in C/T and T/T genotypes compared to controls (Figure 2). In contrast, in the Bulgarian control groups, we only observed an increase in the homozygous T/T genotype, although these differences did not reach statistical significance at the α < 0.05 level. These results suggest that homozygous T/T carriers of the CHGB P413L variant may be associated with an increased susceptibility to ALS in the Bulgarian population. The most striking difference was observed in the comparison with NFE controls (χ^2^ = 15.4572, *p* = 0.000440). This pattern across both allelic and genotypic analyses strengthens the evidence for a potential role of the CHGB P413L variant in ALS susceptibility. However, our findings contrast with some previous studies, as previous reports for Italian and French cohorts reported no association for the variant [32,33]. These discrepancies highlight the potential for population-specific effects and underscore the importance of studying genetic variants in diverse populations, as the variant’s frequency ranges from 25.84% in admixed American, to 1.9% in European Finnish (data from GnomAD v.2.1.1 [42]). It could also suggest that there are different multifactorial pathological drivers of ALS, which can vary between cohorts.

However, our investigation into the modifier effect of the P413L variant on age of onset yielded unclear results. The one-way ANOVA analysis did not reveal statistically significant differences in age of onset across the three genotypes (C/C, C/T, and T/T) or between carriers and non-carriers of the T allele. The mean age of onset for C/C homozygotes was 58.35 years, compared to 55.27 years for C/T heterozygotes and 59.50 years for T/T homozygotes. While there appears to be a trend towards earlier onset in C/T heterozygotes, the small sample size of T allele carriers (particularly T/T homozygotes, *n* = 2) limits the statistical power of this analysis. The Kaplan–Meier survival curves provided a more nuanced view of the potential impact on age of onset. At the genotype level, C/T heterozygotes showed a trend toward earlier onset compared to C/C homozygotes, while T/T homozygotes had a later mean onset. At the allele level, carriers of the T allele displayed a mean onset age 2.5 years earlier than non-carriers. This suggests a possible dosage effect, where a single T allele may be associated with earlier onset. However, the limited number of T/T homozygotes in our cohort necessitates a cautious interpretation of this finding. The narrower age range observed for T allele carriers (33–70 years) compared to non-carriers (28–81 years) is intriguing and may suggest a more constrained window of disease onset for carriers. This could potentially reflect a specific pathogenic mechanism associated with the variant, although the limited sample size could be a better explanation.

Our findings contribute to the ongoing debate regarding the role of the CHGB P413L variant in ALS. Our results, showing a significant association with ALS susceptibility but an unclear effect on age of onset, highlight the complex nature of genetic influences in ALS and the potential for population-specific effects. The increased frequency of the T allele in our ALS cohort suggests that the CHGB P413L variant may indeed play a role in ALS pathogenesis. Given the known functions of CHGB in neurotransmitter release, protein homeostasis, and ion channel formation, it is plausible that this variant could contribute to ALS through multiple mechanisms. For instance, the variant might affect the sorting of CHGB into secretory granules, potentially disrupting neurotransmitter release and contributing to motor neuron dysfunction. Additionally, alterations in CHGB function could impact ion homeostasis and protein folding, processes known to be disrupted in ALS [39,40]. Furthermore, as CHGB forms anion channels that are delivered to the cell surface via regulated secretion [38], alterations in these channels due to genetic variants could potentially contribute to the ion imbalances observed in ALS. The variant itself could impair the channel’s conductivity, as well as have an impact on channel formation and export to the cell membrane.

However, the lack of a clear effect on age of onset in our study suggests that the impact of this variant may be more subtle or complex than previously thought. It is possible that the P413L variant acts as a risk factor for ALS development but does not significantly influence disease progression once initiated. Alternatively, its effects on age of onset may be modulated by other genetic or environmental factors not captured in our study. Such limitations of our study include the relatively small sample size, particularly for T allele carriers, which limits the statistical power for detecting subtle effects on age of onset. Additionally, our focus on a Bulgarian population, while valuable for understanding population-specific effects, may limit the generalizability of our findings to other populations.

Future research should further investigate the impact of the CHGB P413L variant on oxidative stress and its role in the pathogenesis of ALS. CHGB is known to play a significant role in the regulation of oxidative stress pathways, and recent evidence suggests that variations in CHGB could disrupt the balance of reactive oxygen species (ROS) within neurons, contributing to neurodegeneration observed in ALS [44]. Another option for further studies of this variant is how influences oxidative stress markers, such as isoprostanes [45] and other lipid peroxidation products, to elucidate its contribution to ALS progression. This could lead to the identification of novel therapeutic targets aimed at restoring oxidative balance in affected neurons. The variant’s role in RNA stability, particularly concerning the expression of neuroendocrine genes implicated in ALS, as well as proteomic analyses in cellular and animal models expressing the P413L variant will be essential to uncover the molecular mechanisms at play.

Furthermore, the CHGB P413L variant presents a promising candidate as a biomarker for ALS, especially within specific populations, where this variant can appear with higher frequency. The strong association between CHGB variants and oxidative stress pathways suggests that this variant could be utilized to predict ALS progression and patient outcomes more accurately. Secretogranin-1, the protein encoded by CHGB, has been found in cerebrospinal fluid in patients with Alzheimer’s disease and these levels correlate with amyloid β and amyloid precursor protein levels in cerebrospinal fluid (CSF) [46]. Monitoring oxidative stress markers alongside CHGB levels in CSF or blood may enhance early diagnosis and disease monitoring, offering a more nuanced approach to ALS management [47]. Moreover, if the P413L variant influences RNA stability, protein synthesis or channel formation, it can also be a target for personalized therapeutic strategies, potentially leading to better-targeted interventions that specifically address the molecular disruptions caused by the P413L variant in patients with ALS.

In conclusion, our study provides evidence for an association between the CHGB P413L variant and ALS susceptibility in the Bulgarian population, while its effect on age of onset remains unclear. These findings underscore the importance of population-specific studies in understanding the genetic basis of ALS and highlight the need for further research with larger cohorts and diverse populations to fully elucidate the role of CHGB in ALS pathogenesis. Future studies should also consider potential interactions between the CHGB P413L variant and other genetic and environmental factors that may influence ALS onset and progression.

## Figures and Tables

**Table 1:** Counts and age of onset mean, minimum and maximum for the patient cohort.

	Males	Females
Count	85	65
Mean Age of Onset	56.88 ± 12.83	60.4 ± 10.47
Minimum Age of Onset	28	37
Maximum Age of Onset	81	79

**Table 2:** Results from the chi-square tests performed on the patient cohort. NFE—non-Finnish European. Statistically significant *p*-values are shown in bold.

**Genotype Data**								
**Comparison Group**	**χ^2^**	***p*-Value**	**Control C/C**	**ALS C/C**	**Control C/T**	**ALS C/T**	**Control T/T**	**ALS** **T/T**
Bulgarian Control	1.9395	3.79 × 10^−1^	91.45%	91.33%	8.26%	7.33%	0.29%	1.33%
Bulgarian Non-Neuro	3.1443	2.08 × 10^−1^	91.59%	91.33%	8.41%	7.33%	0.00%	1.33%
NFE Control	15.4572	**4.40 × 10^−4^**	93.06%	91.33%	6.81%	7.33%	0.13%	1.33%
NFE Non-Neuro	13.7498	**1.03 × 10^−3^**	92.64%	91.33%	7.21%	7.33%	0.15%	1.33%
**Allele Data**								
**Comparison Group**	**χ^2^**	***p*-Value**	**Control C**	**ALS C**	**Control T**	**ALS T**		
Bulgarian Control	0.0531	8.18 × 10^−1^	95.58%	95.00%	4.42%	5.00%		
Bulgarian Non-Neuro	0.1126	7.37 × 10^−1^	95.80%	95.00%	4.20%	5.00%		
NFE Control	1.458	2.27 × 10^−1^	96.46%	95.00%	3.54%	5.00%		
NFE Non-Neuro	0.9571	3.28 × 10^−1^	96.24%	95.00%	3.76%	5.00%		

## Data Availability

Genotyping data for the examined patient cohort are available on request. Allele frequency data were obtained through GnomAD 2.1.1. (gnomad.broadinstitute.org).
